# Gynecologecal and Obstetrical Society of Baltimore

**Published:** 1891-08

**Authors:** William S. Gardner

**Affiliations:** Secretary, 112 N. Howard, St.


### GYNECOLOGECAL AND OBSTETRICAL SOCIETY OF BALTIMORE.

### MAY MEETING.

The President, Dr. Henry M. Wilson, in the chair.

DR. BRINTON read a paper enttiled "A day's Work in Obstetrics." Under this title he related the following cases.

1. A case of podalic version.
2. A case of normal labor.
3. A case of Shoulder Presentation;

Efforts at version unsuccessful; vagina ruptured; the woman dying undelivered.

4. A case of Placenta Previa Lateralis treated by Internal Podalic version, mother and child saved.

DR. MILTENBERGER: There is some discussion in regard to the preference for high forceps and version. I prefer version, but the profession is divided and the choice comes to a matter of skill and individual practice.

DR. NEALE: One of the points claimed for version over high forceps is that in version the narrower diameters of the head come first. It has been claimed that the same condition is brought about in the use of forceps by the diminution of the diameters of the crown, so that they are less than those of the base of the skull. I can not see how this is, for certainly the forceps do not as a rule compress sufficiently to reduce the diameters of the crown to less than those of the base of the head.

Repeated attempts at version has often given bad results when the uterus is contracted and retracted when there is a neglected cross birth and the child is dead. After a moderate attempt at version has failed decapitation should be done.

By means of Brown's Hook it is certainly a comparatively easy and safe procedure.

I have no criticisms to make upon the treatment Dr. Brinton adopted in his cases.

DR. BRINTON: Since this case of rupture of the vagina has been reported, it has been stated by a pathologist of this city that it is the only one on record. I would like to ask if any of the gentlemen present know of any such cases?

DR. MILTENBERGER: There are certainly on record many cases of rupture of the vagina. I have seen at least two such cases.

DR. THOS. A. ASHBY: I once passed a sound through the uterus. The sound went in easily, and could be felt just below the umbilicus. Before this the patient had had pus running slowly from the uterus which had evidently had it's origin higher up. There were no bad symptoms; the woman rode home a distance of eight miles and is not heard from.

I once attempted to remove an epithelial growth from the vagina and all at once the intestines came down. I cleaned away the diseased tissue, closed up the opening with a firm stitch and the wound healed promptly. The patient lived eleven montbs.

DR. GEO. W. MILTENBERGER read a paper upon "Superfoetation and Superfecundation."

DR. P. C. WILLIAMS: I had a case recently of ovulatons during lactation. A lady came to me who had continued to nurse her child and is now five months pregnant. These cases show that there may be ovulation without menstruation, and led me to agree with Dr. Miltenberger.

DR. ASHBY: I have had cases similar to Dr. Williams. I have been surprised at the frequency with which menstruation returned after apparent removal of both ovaries and tubes. One of the first cases upon which I operated, was one of hystero epilepsy. I thought I had removed all the ovarian tissue, but found subsequently that I had not. She began to menstruate about eight months after the operation, and afterwards suffered from menorrhagia. Three years later I examined her under chloroform and found a small tumor. I operated and removed a small portion of an emptied ovary. She recovered promptly and has not menstruated. Her health is good and there has been no return of the hystero-epilepsy. I have had other cases in which some parts of the ovaries had been left behind. These women continued to menstruate. In those cases where I have succeeded in removing the ovaries entirely. I have not observed the return of menstruations.

DR. B. B. BROWNE; I attended a woman a few years ago who had had seven children and had never menstruated. She was married before menstruation began and had had children very frequently.

I think Superfoetation does occur. It certainly occurs in uterus septus.

The removal of the ovaries has little to do with the cessation of menstruation, but the tubes have much to do with it, and it is when a portion of the tube remains behind that menstruation continues. Menorrhagia will occur when the tube is closed at the outer extremity. When a part of the ovary is left, of course a part of the tube is left also.

DR. W. E. MOSELY: My experience has been such as to make me believe that menstruation does not depend upon the presence of the Fallopian tubes, nor is it independent of the ovaries. Eighteen months ago I opened a lady's abdomen for a very severe case of chronic Pelvic Peritonitis with double Pyosalpinx. Both tubes were tied close to the uterus and resume, but after a diligent search no trace of either ovary could be found. Dr. W. H. Welch, to whom the specimens were shown, expressed the opinion that the ovaries had probably been destroyed in the inflammatory process. The patient made a good recovery after very prolonged drainage, made necessary by the sloughy condition of the pelvic contents and the fecal fistula, which persisted for several weeks. This patient for months has been menstruating regularly and freely every three weeks. In all probability some portion of ovarian tissue escaped destruction.

In another case in which I took special pains to remove every particle of each ovary and both tubes on account of severe hemorrage, the patient has not had a show during the past twelve months.

DR. ASHBY: Mr. Tait has maintained the position of Dr. Browne for several years.

In one case the patient had been suffering from hemorrage of tubal origin; I removed both tubes and one ovary. The other having undergone cystine degeneration it was impossible to remove all the ovarian tissue. This patient has been cured of her metrorrhagia but has a vernal menstruation.

DR. OPIE: It seems quite well established by post mortem results, that all cases of menstruation following oophouctomy are not due to failure on the part of the surgeon to completely remove the ovaries.

The utero-ovarian ligament however, is sometimes very short, and the button-like section beyond the ligature which in such cases contains ovarian stroma, may keep up a dominating influence; again the anatomical shape of the ovary gradually sloping off into the ligament, causes a part of the ovarian tissue to be left on the uterine side of the ligature in spite of the utmost care on the part of the operator.

The rule after child-birth seems to be that menstruation is in abeyance for a variable number of months, but cases have doubtless occurred in the experience of most obstetricians, when it has been uninterrupted during lactation. I have met with a number of cases when women have conceived during lactation, when there was no accompanying monthly flow. Dr. Tait thinks that during and even after the menopause, ovulation goes on, though the mucous membrane is disqualified for securing a fecundated ovale. Ovulation may be going on during lactation but the mucous lining of the uterus may not be well qualified for menstruation or fecundation.

Dr. Bush, of New York, who has a dairy farm, has been performing some interesting experiments, to find out the mode of securing the best quality of milk. He has determined that the heifer after the removal of the ovaries can be made a perpetual milker and that the milk is of a better quality than in cows subject to ovulation and impregnation.

DR. BRINTON: With reference to menstruation after the removal of ovaries, we have the statement that one or two per cent. of women have supernumerary ovaries, and possibly the return of the menstruation is due to the presence of the third ovary.

DR. MILTENBERGE: Dr. Brown laid muoh stress upon the fact that menstruation continued when the obstructed tubes were present. Menstruation has nothing to do with the passage of the ovule along the tubes, but is due to the maturation of the ovule.

Therefore the tube may be obstructed as much as you please and there will be no results. Battey and Engleman have reported a number of cases of pregnancy after the ovaries were apparently removed by skillful operators. In other cases the ovaries supposed to be removed, have been found post mortem.

DR. BROWN: In most cases where the ovary and tubes are removed the lumen of the tube is obstructed by the ligation.

DR. ASHLEY exhibited a specimen of a ruptured Tubal Pregnancy which he had removed from a patient seen in consultation with Dr. Arthur Williams, of Elk Ridge, Md. The patient was 34 years of age and gave birth to one child ten years ago. She conceived in February of this year, and about the eighth week of gestation was seized with violent symptoms of intra pelvic hæmatocele. Dr. Williams was called in and after examination diagnosed the condition as a ruptured tubal pregnancy. I saw the patient with him the following day, and upon examination confirmed the diagnosis. The patient rallied from the shock of the first rupture and one week later a second rupture took place, though not followed with such violent and dangerous symptoms as in the first instance. The surroundings of the patient were so unfavorable that she was removed from her home in Anne, Arundel Co., to the Md. Gen'l. Hospital, where the laparotomy was performed. Upon opening the abdomen her pelvic was filled with bloody urine, blood clots and evidences of general peritonitis. The omentum was in such a condition that it was found necessary to remove about three-fourths of the tissue.

The patient was critically ill from the third to the fifth day from symptoms of intestinal obstruction. Her bowels were moved by administering one-grain doses of calomel every hour for twelve hours, every other method having failed. The patient has made a successful recovery.

This is the third case of tubal pregnancy I have removed by laparotomy within the past two years, all of them having recovered. WILLIAM S. GARDNER, M. D., Secretary,

112 N. Howard St.

